# The Effect of Corneal Thickness, Densitometry and Curvature on Intraocular Pressure Measurements Obtained by Applanation, Rebound and Dynamic Contour Tonometry

**DOI:** 10.3390/vision4040045

**Published:** 2020-10-21

**Authors:** Marco Antonio de Castro Olyntho Junior, Lucas Bertazzi Augusto, Carolina P. B. Gracitelli, Andrew J. Tatham

**Affiliations:** 1 Olyntho Oftalmo.center, 15091-751 São José do Rio Preto, São Paulo, Brazil; augusto@clinicaolyntho.com.br; 2 University of Edinburgh, Edinburgh EH8 9YL, Scotland, UK; andrewjtatham@gmail.com; 3 Department of Ophthalmology and Visual Sciences, Federal University of São Paulo, 13565-905 São Paulo, Brazil; carolepm@gmail.com; 4 Centro de Estudos Alcides Hirai, Ver Mais Oftalmologia, 07750-000 Vinhedo, São Paulo, Brazil

**Keywords:** corneal densitometry, tonometry, intraocular pressure, glaucoma

## Abstract

Evaluate the effect of corneal thickness, densitometry and curvature on intraocular pressure (IOP) measurements obtained by Goldmann applanation tonometry (GAT), non-contact tonometry (NCT), rebound tonometry (RT), and dynamic contour tonometry (DCT). A cross-sectional prospective study involving 40 participants was performed. Corneal measurements were obtained using Pentacam (Oculus GMbH, Wetzlar, Germany), densitometry was measured at annuli of 0–2, 2–6, 6–10 and 10–12 mm. The relationship between corneal thickness (central, 4 and 6 mm), corneal astigmatism and corneal densitometry and IOP was examined. There was a significant relationship between corneal thickness (central, 4 and 6 mm) and GAT180, GAT90, RT, and NCT (*P* < 0.001 for all comparisons) but not for DCT. Higher corneal densitometry (6–10 mm and 10–12 mm zones) was associated with higher IOP from GAT180 and GAT90, and higher densitometry in the 6–10 mm zone correlated with higher IOP from NCT, however corneal densitometry increased with age. Accounting for age, the relationship between corneal densitometry and IOP measurements was not significant. In eyes with greater corneal astigmatism there was a greater difference between GAT90 and GAT180 measurements. IOP measurements may be affected by corneal thickness, densitometry and curvature. DCT was less affected by properties of the cornea compared to other devices.

## 1. Introduction

Raised intraocular pressure (IOP) is a major risk factor for the development and progression of glaucoma and therefore accurate IOP assessment is essential to optimal glaucoma management [1,2,3]. However, as IOP is measured through the cornea, readings are affected by characteristics of the cornea such as corneal curvature, thickness and biomechanics [4,5]. Measurements of central corneal thickness (CCT) and corneal biomechanical properties such as corneal hysteresis provide useful additional information for risk profiling but also have a confounding effect on the measurement of IOP [2,6,7,8,9,10].

Goldmann applanation tonometry (GAT) remains widely regarded as the gold standard method of IOP measurement [6,11,12,13,14,15], but a range of newer devices are available including non-contact tonometry (NCT), rebound tonometry (RT) and dynamic contour tonometry (DCT) [6,7,8,9,11,16,17,18,19,20,21,22,23,24,25,26,27,28]. NCT uses an air puff that deforms the cornea, with the advantage of not requiring anesthetic eye drops [8,21,26,29]; RT assesses IOP by firing a small plastic-tipped probe at the surface of the cornea, with the speed at which the probe returns to its initial position providing information on IOP [22,23,30,31,32]; and DCT uses a slit lamp mounted device containing a pressure sensor. DCT has previously been shown to be less affected by properties of the cornea than GAT [15,18,33].

Several studies have examined the relationship between CCT and IOP measured with different devices, however, these have largely relied on ultrasound pachymetry limited to the central cornea [6,16,18,23,24,26,31,34,35,36,37]. Some tonometers such as RTs come into contact with only a small area of the cornea and as corneal thickness varies considerably in different regions, it is important to evaluate whether more eccentric measurement of the cornea may impact IOP assessment [3,12,29,30,31,36,37,38,39,40,41,42,43]. Also, many previous studies have failed to take account of variation in corneal curvature (i.e., astigmatism) which is known to affect the accuracy of GAT measurements [20,26].

Scheimpflug densitometry is an objective, non-invasive method of measuring corneal density and thickness, providing quantitative maps with excellent repeatability [5,44,45,46,47,48]. Scheimpflug devices also provide information on corneal curvature across the corneal surface. The density of the cornea can be altered by diseases of the cornea such as keratoconus [49,50,51] and by refractive surgical procedures [44]. Corneal densitometry also increases with age [5,46,49,52]. However, to the best of our knowledge, no previous studies have examined the relationship between corneal densitometry and IOP and the potential confounding effect of corneal densitometry on the relationship between IOP and corneal thickness and corneal curvature.

This study aimed to further understanding of the effect of corneal thickness, corneal densitometry and corneal curvature on IOP measurements obtained using different devices. The relationship between corneal thickness, keratometry and density measured using Scheimpflug imaging, and IOP measurements obtained from GAT, RT, NCT and DCT were examined.

## 2. Materials and Methods

This was a cross sectional prospective study including 40 consecutive patients attending the Olyntho oftalmo.center, São Paulo, Brazil. The study adhered to the tenets of the Declaration of Helsinki and was approved by CEP-CONEP Kayser Clinica and Hospital Dia (number: 3.583.006). In addition, written informed consent was obtained from each participant before any examination. 

All participants had a complete medical history and ophthalmologic examination including uncorrected and corrected visual acuity, static and dynamic refraction, slit-lamp examination, gonioscopy and dilated fundoscopy. Data collected include age, sex, race and history of ocular disease or surgery.

Exclusion criteria included any abnormalities of the cornea, such as opacities, oedema and dystrophy, as well as patients who had undergone any previous corneal surgical or laser procedure. Patients who received chronic ocular medication or systemic medication that may have induced changes to the cornea or ocular surface were also excluded, for example, those using amiodarone. 

### 2.1. Corneal Measurements

Anterior segment images were obtained using the Pentacam (Oculus GMbH, Wetzlar, Germany) (Software v. 1.21r43 [Build 5293]). Pentacam allows evaluation of the cornea and anterior chamber using a Scheimpflug rotational camera. The equipment performs image acquisition through a rotational camera that scans the entire anterior segment of the eye, while a second camera analyses the fixation of the patient’s eye. Twenty-five thousand distinct corneal elevation points are examined, with a series of 25 images (1003 × 520 pixels) acquired in 2 s, and with the images obtained, it is possible to recreate a 3D image of the anterior segment. 

**Figure 1:**
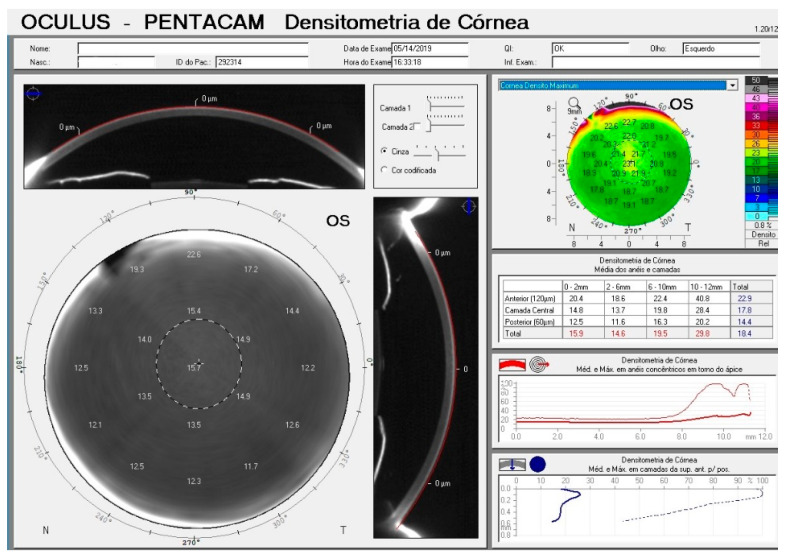
Pentacam Densitometry map for the left eye of a patient included in the study.

Measurements of the optic density of the cornea were obtained by analysis of light scattered over the corneal surface and the inner layers. Measurement was presented on a standard scale of 0 to 100 greyscale units (GSU), where 0 defines the minimum light scattering (maximum transparency) and 100 the maximum light scattering (minimum transparency). An example of a corneal density map obtained is shown in Figure 1. 

The system presents images with sectional planes of 90 and 180 degrees, and the distribution of total measurement points distributed in several measurements from the apex of the cornea to its periphery, in grayscale. A colored map shows the points collected from the densitometry at each point of the cornea measured within the 12 mm zone distributed into four concentric radial zones, with values ranging from 0 to 2, 2 to 6, 6 to 10 and 10 to 12 mm. Values are presented in annular measurements from the apex of the cornea in 3 different layers of the cornea, the first 120 is corresponding to the anterior portion of the cornea, following the central stroma, and later to the last 60 of the cornea, Descemet’s membrane and endothelium. The Pentacam also provided information on CCT and K1 / K2 keratometry readings. Corneal astigmatism was calculated for each eye as the difference between K1 and K2 readings. All examinations were performed in a nonmydriatic state and before IOP measurements or instillation of topical eye drops.

### 2.2. Intraocular Pressure Measurements

IOP was measured using GAT, NCT, RT and DCT, with the order of testing randomized and a maximum interval of 5 min between each measurement. NCT was performed with an air tonometer TRK-1P (Topcon Co, Tokyo, Japan). IOP was determined from the average of 3 measurements, with measurements repeated if there was discordance of greater than 3 mm Hg. 

Rebound tonometry (RT) was performed with the iCare Pro (TA03) (iCare Finland Oy, Vantaa, Finland), with the patient in a sitting position. IOP was determined as the mean of six measurements taken in rapid succession and only measurements with a standard deviation of less than 15% were considered. If the standard deviation was greater, measurements were repeated. 

Dynamic contour tonometry (DCT) was carried out using Pascal DCT (SMT Swiss Microtechnology AG, Port, Switzerland). DCT has an automatic calibration system and measurement is performed by touching the central cornea with the concave 10.5 mm radius of curvature tip with a sensor protected by a disposable silicone cover, performing around 100 transcorneal measurements per second. DCT was performed after a drop of 0.5% topical proparacaine hydrochloride (Anestalcon, Alcon Laboratories, Inc., Fort Worth, TX, USA). DCT measurements were required to have a quality value of Q1 or Q2. Goldmann Applanation Tonometry was performed using the AT 900 (Haag-Streit, Koeniz, Switzerland) [7,8,11,12,13,14]. Before each measurement, topical anesthetic eye drops 0.5% topical proparacaine hydrochloride (Anestalcon, Alcon Laboratories, Inc., Fort Worth, TX, USA) and fluorescein eye drops (Fluoresceina Sódica, Ophthalmos, SP, Brazil) were instilled in each eye. 

GAT IOP was performed with the prism on axis 180 (GAT 180) and axis 90 (GAT 90), measured three times in each position, with the probe moved away from the cornea between each measurement, and the mean IOP recorded in the chart. NCT and RT were performed by 2 experienced technicians and DCT and GAT by an ophthalmologist. Examiners were masked to the results of previous tests, and IOP measurements were performed with a minimum interval of 5 min between each equipment to avoid any interference. 

### 2.3. Statistical Analysis

Descriptive statistics included mean and standard deviation for normally distributed variables and median, quartiles for those non-normally distributed. The Kolmogorov–Smirnov normality test was performed to assess the normality of the distribution. The relationship between corneal densitometry obtained by Scheimpflug imaging in several areas of the cornea and IOP measurements obtained from the four tonometers was examined using scatter plots and regression analysis. Similar analyses were performed to examined possible relationships between IOP other parameters including CCT, corneal curvature (CR), spherical equivalent (SE) and corneal astigmatism. Spearman’s correlation coefficient (rho) was used for non-parametric variables and Pearson Correlation Coefficient was used for parametric variables. Statistical analyses were performed with commercially available software (STATA, v. 13; StataCorp LP, College Station, TX and SPSS “Statistical Package for Social Science for Windows” SPSS Inc., v. 24). The alpha level (type I error) was set at 0.05.

## 3. Results

The study included 40 healthy participants with a median age of 39.0 years (interquartile (IQ) range, 32.0 to 49.0). Twenty-six (32.5%) were female, 36 (90%) were White, 3 (7.5%) Asian and 1 (2.5%) Black. The demographic and clinical characteristics of included subjects are summarized in Table 1. 

Median GAT IOP was 13 mm Hg (IQ range 11 to 15 mm Hg) at 180 degrees and 13 mm Hg (IQ range 12 to 15 mm Hg) at 90 degrees. On average IOP was higher using RT (median 15.2 mm Hg (IQ range 12.8 to 16.9 mm Hg)) and using NCT (median 16.0 mm Hg (IQ range 14.4 to 18.5 mm Hg)), whereas DCT IOP was slightly lower (median 12.1 mm Hg (IQ range 10.5 to 13.6 mm Hg). Median CCT was 530.5 µm, increasing to 541.5 µm at 4 mm and 573.5 µm at 6 mm. 

The mean differences and 95% limits of agreement for the IOP devices are shown in Table 2. The smallest differences were observed for GAT180 and GAT90 (mean difference of 0.9 mm Hg lower IOP with GAT90, 95% limits of agreement of −4.1 mm Hg to 2.4 mm Hg). Poorest agreement was between NCT and DCT, with a mean difference of 3.9 mm Hg lower IOP measured with DCT (95% limits of agreement of −11.1 to 3.2 mm Hg). 

### 3.1. Corneal Thickness and Densitometry

There was significant correlation between corneal thickness in the central, 4, and 6 mm zones and GAT 180, GAT 90, RT, and NCT (*P* < 0.001 for all comparisons, Table 3).

**Figure 2:**
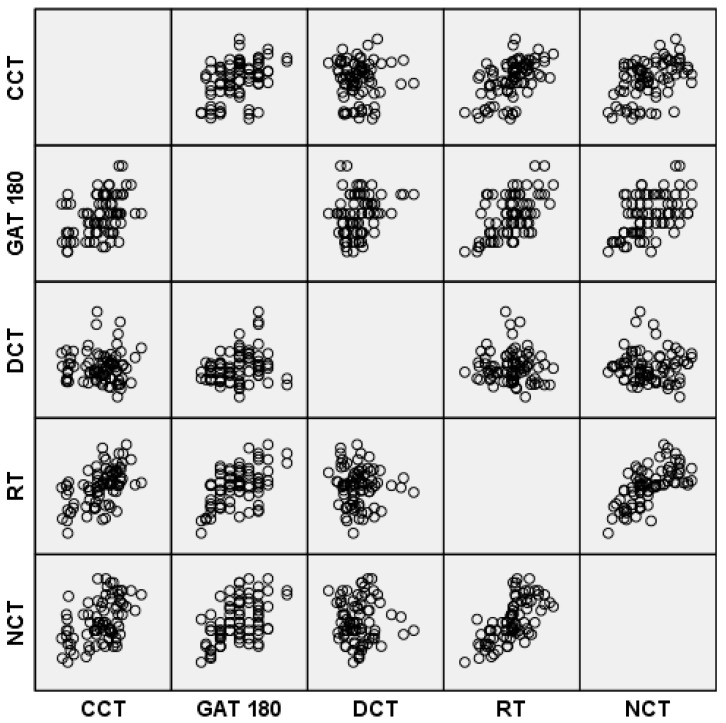
Scatterplots showing the relationship between IOP measurements from GAT, DCT, RT and NCT and central corneal thickness (CCT) and the relationship between IOP measurements from each device.

In contrast, there was no correlation between DCT IOP measurements and any of the corneal thickness measurements. In the scatterplot graph, it is possible to analyze the correlation of IOP measurements with the central corneal thickness (Figure 2). There was also positive correlation between CCT and corneal thickness in the 4 and 6 mm zones and with SE. 

Median corneal densitometry readings were 16.1, 14.8, 17.4 and 22.7 GSU at 0–2, 2–6, 6–10 and 10–12 mm respectively (Table 1). There was significant correlation between higher corneal densitometry values in the 6–10 and 10–12 mm zones and higher IOP readings from GAT180 and GAT90, and between corneal densitometry in the 6–10 mm zone and NCT, however there was no correlation between other IOP and corneal densitometry comparisons.

**Figure 3:**
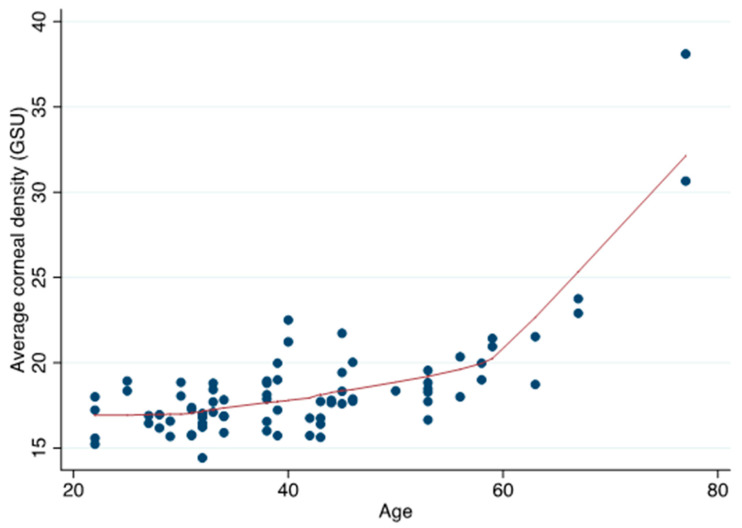
Scatter plot showing the relationship between age and average corneal densitometry (GSU).

There was a significant increase in corneal densitometry values with older age, indicating a reduction in corneal clarity, at 2–6, 6–10 and 10–12 mm, though not at 0–2 mm (Table 3, Figure 3).

Accounting for age in multivariable regression, the relationship between corneal densitometry and GAT180, GAT90 IOP measurements was no longer significant (Table 4). Accounting for age, the relationship between corneal densitometry in the 6–10 mm zone and NCT IOP was also no longer significant (coefficient for densitometry = 0.004, 95% CI −0.032 to 0.040, *P* = 0.841, coefficient for age = 0.065, 95% CI 0.017 to 0.113, *P* = 0.008).

### 3.2. Refraction and Astigmatism

There was positive correlation between SE and IOP measurements from GAT180, DCT, RT and NCT but not between SE and GAT 90 (Table 3). SE was also positively correlated with age, CCT, astigmatism and corneal densitometry in the 6–10 mm zone. Higher degrees of corneal astigmatism were correlated with older age and higher SE but not IOP (Table 3). There was however correlation between greater absolute differences between GAT180 and GAT90 measurements and greater magnitudes of corneal astigmatism (rho = 0.306, *P* = 0.006). There was no relationship between the difference in GAT180 and GAT90 and the meridian of corneal astigmatism (rho = −0.008, *P* = 0.942). 

As many eyes had low levels of corneal astigmatism, the analysis of agreement between GAT90 and GAT 180 was repeated for eyes with ≥0.5D of corneal astigmatism. For eyes with ≥0.5D of with-the-rule (WTR) astigmatism, there was a mean difference of −1 mm Hg (95% limits of agreement −4.5 to 2.5 mm Hg) compared to a mean difference of 0.2 mm Hg for against-the-rule (ATR) astigmatism (95% limits of agreement −0.7 to 1.1 mm Hg), though there were only 5 eyes with ≥0.5D of ATR astigmatism, compared to 63 with ≥0.5D of WTR astigmatism. Correlation between GAT180 and GAT90 was greater in eyes with ATR astigmatism (rho = 0.9177, *P* = 0.028 versus rho = 0.673, *P* < 0.001). 

## 4. Discussion

Glaucoma is a progressive, irreversible neurodegenerative disease that causes irreversible visual loss. It is estimated that the disease affects 80 million people around the world. The consequences of the disease range from the economic impact on the cost of chronic treatment to the morbidity caused by the impairment of the visual field and vision [1,14].

The incidence is approximately 2% at 40 years, increasing to 4% in whites and 13% in blacks at age 80, and IOP is the main risk that can be modified to control the disease, and clinical evidence has shown that reduction is critical for the management of glaucoma [2].

Since Glaucoma is a chronic degenerative disease, the patient will have a history of ocular pressures that should be evaluated for a lifetime, is paramount that a tonometer measures the pressure with less bias and be reliable [22]. While other factors are still being studied to achieve proper Glaucoma control, accurate IOP assessment becomes extremely important, and reproducibility and elimination of factors that may interfere become clinically important [3,6,21].

There are several devices available on the market for measuring glaucoma, and studies show that lacks a technology to replace the older ones [27]. The basis of the treatment lies in the reduction of IOP to prevent the progression of Glaucoma, which can be performed using eye drops and medications that are generally very effective, and which can be supplemented with the use of laser, fistulizing surgeries or with the use of valves [14].

However, even if the treatment is considered effective, a good portion of the patients continues to suffer the progression of glaucomatous damage, despite controlled IOP. Because of this, studies must consider evaluating other factors that could interfere in advance of the disease besides the IOP study only [14].

The Early Manifest Glaucoma Trial demonstrated that the reduction of IOP by 1 mm Hg in patients with the disease reported a reduction of approximately 10% in the rate of progression, being an important piece of information that underpins the need for accurate IOP measure [2].

Goldmann applanation tonometry (GAT) is considered the gold standard for IOP measurement [2,14,15,27,34,40]. Due to its simplicity and easy integration of the slit lamp since its launch, quickly gained the preference of ophthalmologists. 

Despite presenting reproducible results, the measure undergoes the effects of CCT. The GAT was developed on the basis that the CCT average would be 500 microns, so with CCT above or below this value, the result is no longer accurate [15,22,25,31,34,40]. For some time it was believed that it would be possible, based on the collection of a large number of data from different racial populations, to develop a formula or nomogram that would allow the correction of GAT values, increasing the accuracy for the treatment of Glaucoma, which was found to be unfeasible [14,22,27,34]. 

The initial GAT equation is based on the Imbert–Fick law, assuming that the radius of curvature and stiffness were constant, the eye was a sphere and the aqueous humor being still during the measurement [22,30]. 

The equipment works based on identifying the force required to cause a defined amount of deformation and flattening in the cornea, described as indentation and applanation [6,14,15].

The IOP value is interpreted with the alignment of 2 semicircles that form at the prism interface. The measurement takes into account the momentum and cannot be performed for a prolonged interval [6,14].

The measurements of PIO are influenced by many factors other than CCT, such as hydration, stiffness, hysteresis, corneal curvature, among other factors [15,23,27,30]. 

In thin corneas the GAT underestimate PIO, and in thick corneas the GAT overestimate PIO [15,21,23]. One study identified that IOP changes at 1.5 mm Hg for each 100-micron increase in CCT [2].

The position of astigmatism may influence GAT values, and studies show that with-the-rule astigmatism causes IOP to be underestimated, and in astigmatism, against-the-rule can overestimate IOP. In corneas with keratoconus or irregular astigmatism, the variables may be unpredictable [30].

Non-contact tonometry (NCT) is also called pneumotonograph. The device produces a constant airflow through the probe that is covered by a diaphragm. The pressure behind the diaphragm increases until it opens, and the flow exits reaching the surface of the cornea, causing deformation and flattening, similar to that caused by the GAT. The airflow flattens the cornea, and when the infrared light, emitted by the device, is totally reflected, the IOP is measured [15]. NCT is widely used, being easily operated by technicians, requiring no anesthetic or dye.

Rebound tonometry (RT) overcame numerous limitations of traditional tonometers because it is portable and does not require the use of topical anesthetic. The data collected from the central corneal region are reported as having a significant correlation with the GAT, but according to it, it also suffers from the influences of CCT and other corneal characteristics, overestimating values in corneas above 520 microns and underestimating IOP in corneas with lower thickness [23,30,36,39]. Pressure measurement is performed by the impact-induction principle, where the deceleration of a magnetic solenoid probe weighing approximately 24 mg is calculated by reaching the center of the cornea. 

The probe has a plastic head with 0.9 mm radius. The speed at which the probe reaches the cornea is approximately 0.25–0.35 m/sec, a velocity that is faster than the blink reflex, allowing measurement in patients with poor coordination, disabilities, corneal scars, or even in the supine position [31]. Due to the practicality of the measurement, some models allow self-measurement, which allows the patient to measure his/her IOP with great convenience and identify peaks at times when they would be practically impossible in the doctor’s office [39]. In a study developed by de Bernardo et al. [40], it was observed that RT is significantly correlated with CCT and CR (r = 0.483, *P* < 0.001 and r = 0.550, *P* < 0.001, respectively).

In a study by Ozcura et al. [23], it was reported that IOP increased by 8 mm Hg for each 100 µm increase in CCT. The same study reported that there is no consensus in the literature regarding this relationship.

In our analyses, we find a moderate correlation with RT measurements and CCT, and a scatterplot distribution was dispersed, compromising Linear Regression analyses.

Dynamic contour tonometry (DCT) is a method of contact measurement, non-invasive, based on the Blaise Pascal law of hydrostatic pressure, where the pressure is defined as a uniformly distributed force, acting perpendicularly in all the portions that limit the fluid, allowing the free reallocation of the molecules of the liquids or gases. The DCT’s probe has a piezo-resistive sensor that comes in contact with the surface of the cornea respecting its contour, causing the external pressure equals to the pressure of the eye, then the computer calculates the IOP [14,15,22,30,53]. 

Since the measurement is obtained without applying force to the surface of the cornea, it is estimated that the measurements are less influenced by the biomechanical properties of the cornea and maintain the natural shape of the cornea during measurement [14,22,25,27,30,40].

DCT mainly performs a continuous trans corneal measurement of the fluid column with the anterior chamber, being compatible the results obtained from experiments with human eyes in living patients, who received cannulation with intraocular pressure measurement, being considered the most accurate and independent of the characteristics of the cornea [34].

DCT performs 100 measurements per second and can detect static as well as dynamic pressures [15].

Kniestedt et al. [33] carried out a cadaver eyes study to evaluate the accuracy of measurements obtained with DCT and GAT. The eyes were connected to a manometer and received a known internal pressure. The DCT presented values closer to the actual values, while the GAT presented a lower pressure 4 mm Hg [6].

A sequence of 6 measurements are performed, which are stored in the tonometer, and at the end, the mean of the measurements is calculated [36].

In addition to IOP measurements, DCT also has the advantage of measuring ocular pulse amplitude (OPA), which is the difference between systolic and diastolic blood pressure, which represents the pulse wave produced by the variation in the amount of blood reaching the eye due to the cardiac cycle [14,23,40].

Therefore, it is believed that the measurements obtained in vivo are closer to the real values, even when considered comparisons with measures of GAT corrected in several states of Glaucoma, being considered by several authors as the tonometry that should be considered gold standard in place of the GAT [6,34].

Despite the advantages presented by the DCT, measurements are time-consuming and are more difficult to obtain than with GAT, and many measures of the same eye are rarely required to obtain a constant value. The sound emitted by the device when performing the procedure encourages the patient to remain aligned and still for as long as is necessary [14]. 

The results of this study are in agreement with previous observations of a significant relationship between properties of the cornea and IOP measurements obtained using applanation, rebound and air puff tonometers. The effect of CCT on IOP measurements taken through the cornea is well known, and our results were in agreement with published literature showing significant correlation between thinner CCT and lower IOP measured by GAT [6,30]. There was also correlation between thinner CCT and lower IOP measurements from RT and NCT, with RT found to have the strongest correlation [23,30,31,36,39,40]. In contrast, IOP measurements from DCT were not influenced by CCT. 

The ocular hypertension study (OHTS) demonstrated CCT to be an important predictive factor for the development of glaucoma and measurement of CCT has become an essential component of glaucoma management [6]. Though formulae have been proposed for adjusting IOP for CCT, these are not recommended as the relationship between CCT and IOP is influenced by other properties of the cornea. Properties of the cornea potentially relevant to transcorneal IOP measurement, include hysteresis, viscosity, elasticity, hydration, connective tissue composition and curvature [2,7,8,21,27]. 

Corneal hysteresis has been identified as an important marker of corneal biomechanics, with low hysteresis a risk factor for the development of glaucoma and glaucoma progression. A limitation of the current study was that corneal hysteresis was not measured, instead the focus was on corneal density and curvature. We also examined the relationship between IOP and corneal thickness at more eccentric regions of the cornea but found similar correlation between corneal thickness at 4- and 6-mm zones, and IOP from GAT, NCT and RT. Only DCT measurements were not related to corneal thickness in any zone.

A novel aspect to this study was to examine the potential relationship between corneal densitometry and IOP. Corneal densitometry is defined as the sum of light scattering caused by the corneal epithelium, stroma, and endothelium [5]. In the normal cornea, most light scattering occurs in the anterior superficial layer of the corneal epithelium and in the anterior cornea where keratocyte density is higher [46,50,51,52,53]. In order to preserve the integrity of the corneal surface, Pentacam has been performed before instilling dyes or anesthetics, which could interfere with the light scattering as well as cause changes in corneal thickness [54,55]. The cornea tends to become less transparent with age, in part due to progressive age-related loss of corneal endothelial cells, which are fundamental for maintaining transparency working as a functional barrier and water pump, but also due to age-related corneal changes in the periphery, including senile arch or crocodile shagreen [46,51]. In agreement with other studies, we found significant correlation between increasing age and corneal densitometry, particularly in the more peripheral cornea. There was no relationship between age and densitometry in the central 0–2 mm annulus [5,45,51]. The stronger relationship between age and changes to densitometry in the peripheral cornea is likely due to a higher prevalence of age-related corneal changes in this region. 

To the best of our knowledge, the effect of corneal densitometry on IOP readings from different tonometers has not been evaluated in detail. We found corneal densitometry was most strongly correlated with GAT. There was also a significant relationship between corneal densitometry in the 6–10 mm zone and NCT, however, densitometry seemed to have no effect on DCT and RT. Corneal densitometry was noted to increase with age, and as older patients in this cohort also tended to have higher IOP, the effect of age was examined in multivariable model. Once age was considered, the relationship between corneal densitometry and IOP from GAT was no longer significant. The meaning of this observation is uncertain, but a possible explanation is that age-related increases in corneal densitometry may to lead to increases in IOP measured using GAT, with less effect observed on IOP measurements from other devices. In support of this theory, there was no relationship between DCT IOP measurements and corneal densitometry, and no relationship between DCT and age. However, RT IOP measurements also increased with age but were not correlated with densitometry. 

In addition to examining corneal thickness and densitometry, an analysis of the relationship between IOP and corneal curvature was conducted using measurements obtained from the Pentacam. There was no relationship between magnitude of corneal astigmatism and IOP using any device, however there was a relationship between greater disagreement between GAT90 and GAT180 and higher corneal astigmatism reflecting the importance of considering the impact of corneal astigmatism on IOP measurements from GAT. Overall, there was good agreement between GAT90 and GAT180, with a mean error of only −0.9 mm Hg and 95% limits of agreement of −4.1 to 2.4 mm Hg. However, in eyes with higher corneal astigmatism there was a greater difference, likely due to the distortion of mires that can occur when performing GAT in eyes with high corneal astigmatism. It is for this reason that guidelines, such as those from the European Glaucoma Society recommend that in cases of high astigmatism, the orientation of the GAT prism is aligned to the axis of the minus cylinder, or that IOP is determined from the average of measurements taken in the horizontal and vertical positions.

To investigate the impact of taking average measurements, we examined agreement between the average of GAT180 and GAT90 IOP and DCT and found overall slightly better correlation compared to GAT90 or GAT180 measurements alone (rho = 0.284, *P* = 0.011 for average versus DCT and rho = 0.243 and 0.240 for GAT90 and GAT180 versus DCT respectively). The mean difference between the average of GAT90 and GAT180 and DCT was 0.96 mm Hg (95% limits of agreement = −4.3 to 6.2 mm Hg), which was better than agreement between GAT90 and DCT but worse than for GAT180 and DCT (Table 3). 

As many eyes had low levels of astigmatism, the analysis of agreement between GAT90 and GAT 180 was repeated for eyes with ≥0.5D of corneal astigmatism. For eyes with with-the-rule astigmatism, there was a mean difference of −1 mm Hg (95% limits of agreement −4.5 to 2.5 mm Hg) compared to a mean difference of only 0.2 mm Hg for against-the-rule astigmatism (95% limits of agreement −0.7 to 1.1 mm Hg). Though this implies that agreement between GAT90 and GAT180 may be better in against-the-rule astigmatism, caution is warranted as there were only 5 eyes with ≥0.5D of against-the-rule astigmatism. 

Previous studies have reported GAT to underestimate IOP in patients with with-the-rule astigmatism and overestimate in those with against-the-rule astigmatism overestimation [30]. We investigated this by examining the difference between GAT180 and DCT measurements in eyes with WTR and ATR astigmatism, and found a GAT180 to overestimate IOP in both groups, however, there was greater overestimation in the ATR group (median overestimation of 1.6 mm Hg (IQ range 0.3 to 3.3 mm Hg)) compared to WTR group (median overestimation of 0.5 mm Hg (IQ range 1.1 mm Hg underestimation to 2.3 mm Hg overestimation). 

Previous studies comparing DCT and GAT have generally shown DCT to produce higher readings that GAT, with DCT less affected by properties of the cornea [2,14,22,25]. For example, Kniestedt et al. [33] carried out a cadaver study to evaluate the accuracy of measurements obtained with DCT and GAT, where the eyes were connected to a manometer. The DCT provided measurements closer to manometric IOP, with GAT tending to underestimate actual IOP by an average of 4 mm Hg [6]. However, others have shown higher IOP values using GAT, with the differences between studies likely due to differences in the characteristics of patients in each study, for example, differences in corneal biomechanics [2,21]. Previous studies examining RT have reported a tendency for RT to overestimate IOP by an average of 2 to 3 mm Hg [3,23,36,42], however some have found RT to underestimate relative to GAT [31], especially in patients with CCT outside 500 to 600 µm [40]. RT seems to be had better agreement with GAT at normal levels of IOP [8,31,40]. Table 3 shows that on average DCT measurements tended to be lower than GAT180, GAT90 and RT, but higher than NCT, whereas GAT180 tended to be lower than GAT90, NCT and RT, but not DCT. 

It is important to acknowledge the limitations of the current study, in particular, the inclusion of only healthy patients with normal corneas and IOP within the statistically normal range. For this reason, it is important not to extrapolate relationships beyond those explored in the study. The study was also limited by a small sample size and failure to examine corneal hysteresis, which is likely to be related to corneal densitometry and corneal shape and is known to have an important cofounding effect on IOP measurements, as well as the possibility of bias due to the randomization of IOP measurements, despite the 5 min space in each measurement. Nevertheless, the results of the study provide useful information regarding the relationship between IOP and properties of the cornea and confirm that of tested tonometers, DCT is the least affected by corneal thickness and corneal densitometry. Corneal astigmatism was not correlated to IOP measurements from any device, however, in eyes with significant astigmatism, GAT measurements are affected by orientation of the tonometer prism. 

## Figures and Tables

**Table 1:** Demographic and clinical characteristics of included subjects.

	Median	Interquartile Range
**Age**	39.0	32.0 to 49.0
**Race**		
**White**	36 (90%)	
**Black**	1 (2.5%)	
**Asian**	3 (7.5%)	
**Spherical Equivalent (D)**	−0.75	−2.25 to 0.75
**K1 (D)**	42.9	41.8 to 44.0
**K2 (D)**	44.5	43.2 to 46.1
**Corneal astigmatism (D)**	1.0	0.60 to 1.70 (range 0.1 to 4.8)
**Corneal Measurements**		
**CCT (µm)**	531	510 to 549
**Pachymetry 4 mm (µm)**	542	518 to 558
**Pachymetry 6 mm (µm)**	575	547 to 589
**Densitometry 0–2 mm (GSU)**	16.1	15.7 to 16.6
**Densitometry 2–6 mm (GSU)**	14.8	14.2 to 15.3
**Densitometry 6–10 mm (GSU)**	17.4	15.1 to 21.4
**Densitometry 10–12 mm (GSU)**	22.7	20.3 to 24.9
**IOP Measurements**		
**GAT 180 (mm Hg)**	13	11 to 15
**GAT 90 (mm Hg)**	13	12 to 15
**DCT (mm Hg)**	12.1	10.5 to 13.6
**RT (mm Hg)**	15.2	12.8 to 16.9
**NCT (mm Hg)**	16.0	14.4 to 18.5

Abbreviations: D: diopter; µm: micron; mm: millimeter; CCT: central corneal thickness; GSU: greyscale unit; Pachy: corneal pachymetry; GAT: Goldmann applanation tonometry; DCT: dynamic contour tonometry; RT: rebound tonometry; NCT: non-contact tonometry.

**Table 2:** Mean differences (and 95% limits of agreement) for intraocular pressure (IOP) measurements obtained by each tonometer.

	GAT180	GAT90	NCT	RT	DCT
**GAT180**		−0.9	−3.4	−2.2	0.5
		(−4.1 to 2.4)	(−8.2 to 1.4)	(−6.8 to 2.4)	(−5.0 to 6.1)
**GAT90**			−2.5	−1.3	1.4
			(−7.1 to 2.0)	(−6.5 to 3.8)	(−4.1 to 6.8)
**NCT**				−1.2	−3.9
				(−5.5 to 3.1)	(−11.1 to 3.2)
**RT**					2.7
					(−4.6 to 10.1)
**DCT**					

**Table 3:** Correlation between IOP measurements using GAT, DCT, RT and NCT, and corneal properties including corneal thickness (central, 4 mm and 6 mm) and corneal densitometry (0–2, 2–6, 6–10 and 10–12 mm).

		Age	CCT	Pach 4 mm	Pach 6 mm	SE	Astigmatism	GAT 180	GAT 90	DCT	RT	NCT	Dens 0–2 mm	Dens 2–6 mm	Dens 6–10 mm	Dens 10–12 mm
**Age**	**Coefficient**		0.064	0.067	0.069	0.716	−0.447	0.359	0.248	−0.161	0.236	0.329	0.125	0.387	0.642	0.394
	***P*-value**		0.285	0.277	0.273	<0.001	<0.001	0.001	0.013	0.076	0.017	0.001	0.135	<0.001	<0.001	<0.001
**CCT**	**Coefficient**	0.064	1.	0.990	0.963	−0.122	0.048	0.418	0.442	−0.036	0.580	0.460	−0.064	−0.053	−0.076	0.017
	**P-value**	0.285		<0.001	<0.001	0.284	0.673	<0.001	<0.001	0.377	<0.001	<0.001	0.286	0.321	0.251	0.442
**Pach4 mm**	**Coefficient**	0.067	0.990	1.000	0.982	0.170	−0.134	0.439	0.469	−0.002	0.568	0.448	−0.051	−0.041	−0.072	0.019
	***P*-value**	0.277	<0.001		<0.001	0.066	0.240	<0.001	<0.001	0.494	<0.001	<0.001	0.326	0.358	0.263	0.435
**Pach6 mm**	**Coefficient**	0.069	0.963	0.982	1.000	0.161	−0.141	0.440	0.451	−0.017	0.552	0.436	−0.122	−0.092	−0.067	0.034
	***P*-value**	0.273	<0.001	<0.001		0.076	0.216	<0.001	<0.001	0.441	<0.001	<0.001	0.140	0.208	0.276	0.382
**SE**	**Coefficient**	0.716	0.201	0.170	0.161	1.000	−0.381	0.226	0.161	−0.268	0.259	0.272	0.013	0.169	0.371	0.110
	***P*-value**	<0.001	0.037	0.066	0.076		<0.001	0.022	0.077	0.008	0.010	0.007	0.453	0.067	<0.001	0.166
**Astigmatism**	**Coefficient**	−0.447	0.048	−0.134	−0.141	−0.381	1,000	−0.184	−0.153	−0.044	−0.168	−0.133	0.026	0.000	−0.179	−0.157
	***P*-value**	<0.001	0.673	0.240	0.216	<0.001		0.105	0.179	0.704	0.138	0.241	0.821	1.000	0.114	0.167
**GAT 180**	**Coefficient**	0.359	0.418	0.439	0.440	0.226	−0.184	1.000	0.703	0.243	0.500	0.492	−0.028	−0.002	0.240	0.279
	***P*-value**	0.001	<0.001	<0.001	<0.001	0.022	0.105		<0.001	0.015	<0.001	<0.001	0.404	0.492	0.016	0.006
**GAT 90**	**Coefficient**	0.248	0.442	0.469	0.451	0.161	−0.153	0.703	1.000	0.240	0.456	0.589	−0.116	−0.008	0.255	0.287
	***P*-value**	0.013	<0.001	<0.001	<0.001	0.077	0.179	<0.001		0.016	<0.001	<0.001	0.153	0.470	0.011	0.005
**DCT**	**Coefficient**	−0.161	−0.036	−0.002	−0.017	−0.268	−0.044	0.243	0.240	1.000	−0.086	−0.024	0.044	−0.061	−0.136	0.079
	***P*-value**	0.076	0.377	0.494	0.441	0.008	0.704	0.015	0.016		0.224	0.417	0.351	0.295	0.114	0.244
**RT**	**Coefficient**	0.236	0.580	0.568	0.552	0.259	−0.168	0.500	0.456	−0.086	1.000	0.690	0.111	0.183	0.125	0.086
	***P*-value**	0.017	<0.001	<0.001	<0.001	<0.001	0.138	<0.001	<0.001	0.224		<0.001	0.163	0.052	0.134	0.223
**NCT**	**Coefficient**	0.329	0.460	0.448	0.436	0.272	−0.133	0.492	0.589	−0.024	0.690	1.000	−0.069	0.040	0.236	0.142
	***P*-value**	0.001	<0.001	<0.001	<0.001	0.007	0.241	<0.001	<0.001	0.417	<0.001		0.271	0.362	0.018	0.104
**Dens** **0–2 mm**	**Coefficient**	0.125	−0.064	−0.051	−0.122	0.013	0.026	−0.028	−0.116	0.044	0.111	−0.069	1.000	0.759	−0.009	−0.052
	***P*-value**	0.135	0.286	0.326	0.140	0.453	0.821	0.404	0.153	0.351	0.163	0.271		<0.001	0.469	0.322
**Dens** **2–6 mm**	**Coefficient**	0.387	−0.053	−0.041	−0.092	0.169	0.000	−0.002	−0.008	−0.061	0.183	0.040	0.759	1.000	0.434	0.164
	***P*-value**	<0.001	0.321	0.358	0.208	0.067	1.000	0.492	0.470	0.295	0.052	0.362	<0.001		<0.001	0.073
**Dens** **6–10 mm**	**Coefficient**	0.642	−0.076	−0.072	−0.067	0.371	−0.179	0.240	0.255	−0.136	0.125	0.236	−0.009	0.434	1.000	0.602
	***P*-value**	<0.001	0.251	0.263	0.276	<0.001	0.114	0.016	0.011	0.114	0.134	0.018	0.469	<0.001		<0.001
**Dens** **10–12 mm**	**Coefficient**	0.394	0.017	0.019	0.034	0.110	−0.157	0.279	0.287	0.079	0.086	0.142	−0.052	0.164	0.602	1.000
	***P*-value**	<0.001	0.442	0.435	0.382	0.166	0.167	0.006	0.005	0.244	0.223	0.104	0.322	0.073	<0.001	

**Table 4:** Multivariable regression analysis examining the relationship between IOP from GAT180 and GAT90 and corneal densitometry, accounting for age.

	Coefficient	95% CI	*P*-value
**GAT180**			
**Age (years)**	−0.27	−0.734 to 0.202	0.261
**Dens 0–2 mm**	0.08	0.042 to 0.115	<0.001
**GAT180**			
**Age (years)**	0.08	0.045 to 0.125	<0.001
**Dens 2–6 mm**	−0.22	−0.606 to 0.127	0.244
**GAT180**			
**Age (years)**	0.07	0.035 to 0.108	<0.001
**Dens 6–10 mm**	0.002	−0.026 to 0.029	0.904
**GAT180**			
**Age (years)**	0.06	0.022 to 0.105	0.003
**Dens 10–12 mm**	0.03	−0.051 to 0.109	0.476
**GAT90**			
**Age (years)**	0.05	0.007 to 0.089	0.023
**Dens 0–2 mm**	−0.36	−0.889 to 0.177	0.187
**GAT90**			
**Age (years)**	0.05	0.002 to 0.094	0.039
**Dens 2–6 mm**	−0.17	−0.606 to 0.271	0.449
**GAT90**			
**Age (years)**	0.04	−0.003 to 0.080	0.068
**Dens 6–10 mm**	0.002	−0.029 to 0.033	0.911
**GAT90**			
**Age (years)**	0.028	−0.020 to 0.075	0.250
**Dens 10–12 mm**	0.04	−0.052 to 0.121	0.395
